# Direct comparison of the next-generation sequencing and iTERT PCR methods for the diagnosis of TERT hotspot mutations in advanced solid cancers

**DOI:** 10.1186/s12920-022-01175-2

**Published:** 2022-02-09

**Authors:** So Young Kang, Deok Geun Kim, Hyunjin Kim, Yoon Ah Cho, Sang Yun Ha, Ghee Young Kwon, Kee-Taek Jang, Kyoung-Mee Kim

**Affiliations:** 1 Department of Pathology and Translational Genomics, Samsung Medical Center, Sungkyunkwan University School of Medicine, #81, Irwon-ro, Gangnam-Gu, Seoul, 06351 Korea; 2 Department of Clinical Genomic Center, Samsung Medical Center, Seoul, Korea; 3 Department of Digital Health, Samsung Advanced Institute of Health Science and Technology, Sungkyunkwan University, Seoul, Korea; 4 Center of Companion Diagnostics, Samsung Medical Center, Seoul, Republic of Korea; 5 Department of Pathology, Hallym University Sacred Heart Hospital, Hallym University College of Medicine, Seoul, Republic of Korea

**Keywords:** *TERT* promoter mutation, PCR, Next-generation sequencing, Comparison

## Abstract

**Background:**

Mutations in the telomerase reverse transcriptase (TERT) promoter region have been proposed as novel mechanisms for the transcriptional activation of telomerase. Two recurrent mutations in the TERT promoter, C228T and C250T, are prognostic biomarkers. Herein, we directly compared the commercially available iTERT PCR kit with NGS-based deep sequencing to validate the NGS results and determine the analytical sensitivity of the PCR kit.

**Methods:**

Of the 2032 advanced solid tumors diagnosed using the TruSight Oncology 500 NGS test, mutations in the TERT promoter region were detected in 103 cases, with 79 cases of C228T, 22 cases of C250T, and 2 cases of C228A hotspot mutations. TERT promoter mutations were detected from 31 urinary bladder, 19 pancreato-biliary, 22 hepatic, 12 malignant melanoma, and 12 other tumor samples.

**Results:**

In all 103 TERT-mutated cases detected using NGS, the same DNA samples were also tested with the iTERT PCR/Sanger sequencing. PCR successfully verified the presence of the same mutations in all cases with 100% agreement. The average read depth of the TERT promoter region was 320.4, which was significantly lower than that of the other genes (mean, 743.5). Interestingly, NGS read depth was significantly higher at C250 compared to C228 (*p* < 0.001).

**Conclusions:**

The NGS test results were validated by a PCR test and iTERT PCR/Sanger sequencing is sensitive for the identification of the TERT promoter mutations.

## Background

Mutations in the telomerase reverse transcriptase (*TERT*) promoter region are frequently observed in specific types of human cancers, leading to enhanced expression of telomerase. Genome-wide association studies have identified multiple variants at the *TERT* locus, which are associated with the lengths of telomeres and risk of several cancers [1, 2] strongly suggesting that this locus is a common susceptibility locus for many human cancers. The most remarkable advancement in improving our understanding of the genetic role of *TERT* in human cancer was the landmark finding of mutations in the promoter region of the *TERT* gene in melanoma using whole-genome sequencing [3, 4]. These mutations have also been reported in other human cancers, such as bladder cancer and glioblastoma [5, 6]. In human cancers, there are two common recurrent mutations in the *TERT* promoter region, which are located at two hotspots: chr5, 1,295,228 (GRCh37/hg19 by Entrez Gene) C>T (C228T) and 1,295,250 (GRCh37/hg19 by Entrez Gene) C>T (C250T), corresponding to the positions 124 and 146 bp upstream of the *TERT* translation start site, respectively [7]. Transcriptional activation of *TERT* via mutation in the promoter region or other mechanisms limits the production of active telomerase in many human cancers [8]. The prognostic power of the *TERT* promoter mutation highlights its potential use as an important biomarker to predict the aggressive clinical behavior in melanoma, glioma, medulloblastoma, bladder cancer, thyroid cancer, urogenital cancer, and laryngeal cancer [9–11]. *TERT* promoter mutation is associated with worse prognosis in melanoma, glioma, meningioma, thyroid cancer, and bladder cancer [12–18] and is also associated with a high risk of malignant transformation and progression to advanced stages in hepatocellular carcinoma [19, 20].

*TERT* promoter mutations in clinical samples are diagnosed using Sanger sequencing and next-generation sequencing (NGS) [21–23]. Recent advancements in DNA isolation and NGS methods have facilitated the sensitive detection of *TERT* mutations in the formalin-fixed, paraffin-embedded (FFPE) tumor tissues. Although only a small percentage (~ 3%) of human DNA is GC rich, the promoter region consists of GC-rich cis-elements [24]. Similarly, the *TERT* promoter region is rich in GC (> 80%), making the DNA of the affected patients less amenable to amplification. Given that the amplification of templates with GC-rich regions is more difficult than those with non-GC-rich regions using the polymerase chain reaction (PCR) [25, 26] and NGS also shows a very low read depth in this region compared to others [27], we attempted to validate the *TERT* promoter mutations detected by NGS with a combination of conventional PCR and Sanger sequencing methods. For this purpose, we used a commercially available iTERT PCR kit to detect the mutations at the two hotspots in the *TERT* promoter region using 103 NGS-verified cases.

## Methods

### Patients samples

In this study, we used a total of 103 cases diagnosed with *TERT* promoter mutations at the C228T and C250T hotspots using the TruSight Oncology (TSO) 500 NGS test in the Department of Pathology and Translation Genomics of Samsung Medical Center between November 2019 and March 2021. To obtain the negative predictive value (NPV), we added 100 *TERT* wild type cancers from colon (n = 34), urinary tract (n = 1), melanoma (n = 4), liver (n = 2), pancreatobiliary tract (n = 17), soft tissue (n = 14), and stomach (n = 28). This study was performed in accordance with the Institutional Review Board guidelines of Samsung Medical Center (IRB 2020-06-045-001) for data analysis and investigational treatments. All patients provided informed consent to participate in this study.

### DNA extraction

Tumors were micro-dissected from most of the samples, except for small samples that were used for the extraction of genomic DNA. Genomic DNA was isolated from the FFPE tissue sections (generally measuring 6–10 mm) and purified using the AllPrep DNA/RNA FFPE Kit (Qiagen, Venlo, Netherlands) [28]. The Qubit dsDNA HS Assay Kit (Thermo Fisher Scientific, Waltham, MA, USA) was used for DNA concentration determination and 120 ng of input DNA was used for library preparation following modification of the manufacturer’s instructions [29]. The DNA integrity number, which is a measure of the size of the DNA fragments and consequently the quality of the DNA, was determined using the Genomic DNA ScreenTape (Agilent Technologies, Santa Clara, CA) on an Agilent 2200 TapeStation system (Agilent Technologies).

### Library preparation, sequencing, and data analysis

A library was prepared using a hybrid capture-based TSO 500 gene library preparation kit (Illumina, San Diego, CA, USA) according to the manufacturer’s instructions. Briefly, the DNA was fragmented using Covaris S2 (Covaris, Woburn, MA, USA) to generate DNA fragments of 90–250 bp, with a target peak of approximately 180 bp. Next, the samples underwent end repair and A-tailing before unique molecular identifier ligation. Then, amplification was performed to add the index sequences for sample multiplexing. Two hybridization/capture steps were performed. Finally, the libraries were pooled, denatured, and diluted to the appropriate loading concentrations. The sequenced data were then analyzed to identify the clinically relevant classes of genomic alterations, including the single nucleotide variants (SNVs), copy number variants, small insertions and deletions (indels), and rearrangements/fusions. In the TSO 500 analysis, unique molecular identifiers determined the unique coverage at each position and reduced the background noise caused by sequencing and deamination artifacts in the FFPE samples. Results of SNVs and small indels with a variant allele frequency (VAF) of less than 2% were eliminated. Data outputs exported from the TSO 500 pipeline (Illumina) [30] were annotated using the Ensembl Variant Effect Predictor (VEP) annotation engine [30], with information from several databases, such as the Single Nucleotide Polymorphism Database (dbSNP), Genome Aggregation Database (gnomAD; genome and exome sequencing), 1000 genomes project database, ClinVar database, Catalogue Of Somatic Mutations In Cancer (COSMIC) database, Reference Sequence (RefSeq) database, and Ensembl and alignment to the hg19 human reference genome GRCh37 version (http://genome.ucsc.edu/). Mutation allele frequencies below predefined thresholds were considered to be wild-type.

### iTERT PCR and Sanger sequencing

PCR was performed using an iTERT Mutation Detection Kit (GENINUS Inc., Seoul, Korea), according to the manufacturer's instructions. The PCR reactions were assembled on ice and preincubated at 94 °C for 15 min, followed by 40 cycles at 94 °C for 20 s, 58 °C for 40 s, 72 °C for 1 min, and a final extension at 72 °C for 5 min using a C1000 Touch Thermal Cycler Kit (Bio-Rad, Hercules, CA). Bidirectional sequencing was performed using the BigDye Terminator v.3.1 Kit (Applied Biosystems, Foster City, CA, USA) on an ABI 3130xL Genetic Analyzer. The results were marked as mutation-positive if a mutation was detected in both the forward and reverse DNA strands [31]. Positive controls were included in each sequencing run: normal human guide DNA (gDNA) (wild-type) and cancer cell (e.g., the C228T‐positive MDA-MG-231 cell line)-derived genomic DNA that yielded the expected *TERT* promoter sequences in each case.

### Statistical analysis

Statistical analyses were performed using GraphPad Prism v.8.0 (GraphPad Software, CA, USA). Visualization of the genetic alterations was conducted using the R-package. All statistical analyses were performed using the SPSS software v.24.0 (IBM Corp., Armonk, NY). The general characteristics and demographic parameters were compared using Fisher's exact test and other quantitative data were analyzed using paired t-tests.

## Results

### NGS with TSO 500

*TERT* promoter mutations were detected in 103 (5.1%) out of 2032 cases and consisted of 79 (77%) C228T, 22 (21%) C250T, and 2 (2%) C228A mutations. Of these 103 cases, the *TERT* promoter mutations were detected in urinary bladder tumor (31/47, 66%), pancreato-biliary (19/127, 15%), hepatocellular carcinoma (22/41, 54%), and malignant melanoma (12/90, 13%). The tumor mutation burden was found to be high in 25 cases with the *TERT* promoter mutations. The precise characteristics of the tumors with *TERT* promoter mutations are shown in Table 1.

**Table 1:** The result of NGS and Sanger sequencing for TERT promoter region

No	Tumor	DNA concentration (ng/μl)	NGS data						Sanger sequencing
			TMB	MSI	NGS	TERT VAF (%)	TERT TD	TERT TV (%)	
1	Liver	20	TMB-low	MSS	C228T	45.6	456	80	C228T
2	Liver	14	TMB-low	MSS	C228T	25.9	201	80	C228T
3	Liver	9	TMB-low	MSS	C228T	25.6	78	70	C228T
4	Liver	31	TMB-high	MSS	C228T	15.4	259	20	C228T
5	Liver	22	TMB-low	MSS	C228T	36.0	114	90	C228T
6	Liver	27	TMB-low	MSS	C228T	41.7	211	80	C228T
7	Liver	9	TMB-low	MSS	C228T	7.7	78	60	C228T
8	Liver	33	TMB-high	MSS	C228T	44.7	94	40	C228T
9	Liver	6.4	TMB-low	MSS	C228T	26.8	112	60	C228T
10	Liver	16	TMB-low	MSS	C228T	34.1	552	60	C228T
11	Liver	11	TMB-low	MSS	C228T	24.7	178	70	C228T
12	Liver	47	TMB-low	MSS	C228T	28.8	66	20	C228T
13	Liver	26	TMB-low	MSS	C250T	27.9	1223	70	C250T
14	Liver	22	TMB-low	MSS	C228T	38.1	578	60	C228T
15	Liver	47	TMB-low	MSS	C250T	12.0	875	60	C250T
16	Liver	35	TMB-low	MSS	C228T	56.2	441	80	C228T
17	Liver	153	TMB-low	MSS	C228T	28.6	398	70	C228T
18	Liver	83	TMB-low	MSS	C228T	24.4	586	80	C228T
19	Liver	135	TMB-high	MSS	C250T	38.2	728	80	C250T
20	Liver	121	TMB-low	MSS	C228T	34.7	254	60	C228T
21	Liver	140	TMB-low	MSS	C228T	20.9	134	70	C228T
22	Liver	139	TMB-low	MSS	C228T	49.4	237	60	C228T
23	Melanoma	50	TMB-high	MSS	C250T	11.4	500	60	C250T
24	Melanoma	98	TMB-low	MSS	C250T	28.8	351	80	C250T
25	Melanoma	52	TMB-high	MSS	C228T	23.8	395	40	C228T
26	Melanoma	76	TMB-low	MSS	C228T	54.6	227	70	C228T
27	Melanoma	76	TMB-low	MSS	C228T	26.9	93	70	C228T
28	Melanoma	190	TMB-low	MSS	C250T	41.5	585	40	C250T
29	Melanoma	138	TMB-high	MSS	C250T	57.5	315	30	C250T
30	Melanoma	49	TMB-low	MSS	C228T	53.4	251	80	C228T
31	Melanoma	195	TMB-high	MSS	C250T	21.7	359	80	C250T
32	Melanoma	137	TMB-low	MSS	C228T	20.8	226	50	C228T
33	Melanoma	138	TMB-low	MSS	C250T	29.3	399	40	C250T
34	Melanoma	136	TMB-low	MSS	C250T	47.5	385	40	C250T
35	Pancreatobiliary	188	TMB-low	MSS	C228T	34.6	81	60	C228T
36	Pancreatobiliary	456	TMB-high	MSS	C228T	20.5	234	50	C228T
37	Pancreatobiliary	50	TMB-low	MSS	C228T	12.1	239	50	C228T
38	Pancreatobiliary	61	TMB-low	MSS	C228T	10.8	249	25	C228T
39	Pancreatobiliary	159	TMB-low	MSS	C228T	11.7	137	10	C228T
40	Pancreatobiliary	15	TMB-low	MSS	C228T	33.1	130	90	C228T
41	Pancreatobiliary	17	TMB-high	MSS	C228T	31.7	145	70	C228T
42	Pancreatobiliary	19	TMB-low	MSS	C250T	16.9	705	40	C250T
43	Pancreatobiliary	57	TMB-low	MSS	C228T	36.6	544	50	C228T
44	Pancreatobiliary	11	TMB-low	MSS	C228T	45.9	270	60	C228T
45	Pancreatobiliary	22	TMB-high	MSS	C250T	18.0	666	60	C250T
46	Pancreatobiliary	45	TMB-low	MSS	C228T	21.7	337	30	C228T
47	Pancreatobiliary	41	TMB-low	MSS	C228T	29.3	246	30	C228T
48	Pancreatobiliary	18	TMB-low	MSS	C250T	12.8	639	60	C250T
49	Pancreatobiliary	23	TMB-high	MSS	C228T	25.1	470	40	C228T
50	Pancreatobiliary	36	TMB-high	MSS	C228T	24.9	503	70	C228T
51	Pancreatobiliary	36	TMB-low	MSS	C228T	19.1	236	40	C228T
52	Pancreatobiliary	11	TMB-low	MSS	C228T	33.1	366	30	C228T
53	Pancreatobiliary	47	TMB-high	MSS	C228T	39.2	199	80	C228T
54	Urinary	20	TMB-low	MSS	C228T	32.6	331	70	C228T
55	Urinary	81	TMB-low	MSS	C228T	22.7	238	70	C228T
56	Urinary	74	TMB-low	MSS	C250T	16.5	388	60	C250T
57	Urinary	172	TMB-high	MSS	C250T	19.7	117	70	C250T
58	Urinary	28	TMB-high	MSS	C228T	32.8	125	80	C228T
59	Urinary	30	TMB-high	MSS	C228T	19.5	41	30	C228T
60	Urinary	32	TMB-high	MSS	C228T	30.9	628	80	C228T
61	Urinary	70	TMB-low	MSS	C228T	21.7	60	60	C228T
62	Urinary	24	TMB-high	MSS	C250T	8.7	69	10	C250T
63	Urinary	35	TMB-low	MSS	C250T	22.3	197	70	C250T
64	Urinary	58	TMB-high	MSS	C228T	37.9	103	60	C228T
65	Urinary	54	TMB-low	MSS	C228T	14.2	106	60	C228T
66	Urinary	31	TMB-high	MSS	C250T	24.7	515	70	C250T
67	Urinary	47	TMB-high	MSS	C228T	28.1	740	70	C228T
68	Urinary	38	TMB-high	MSS	C228T	46.9	260	40	C228T
69	Urinary	43	TMB-low	MSS	C250T	49.2	1140	90	C250T
70	Urinary	25	TMB-low	MSS	C250T	47.6	993	70	C250T
71	Urinary	49	TMB-low	MSS	C228T	34.3	134	40	C228T
72	Urinary	22.4	TMB-low	MSS	C228T	53.8	409	35	C228T
73	Urinary	32	TMB-low	MSS	C228T	10.8	510	50	C228T
74	Urinary	27	TMB-low	MSS	C228T	25.3	301	20	C228T
75	Urinary	63	TMB-low	MSS	C228T	18.4	87	90	C228T
76	Urinary	42	TMB-low	MSS	C228T	47.6	410	70	C228T
77	Urinary	38	TMB-high	MSS	C228T	24.6	272	20	C228T
78	Urinary	63	TMB-low	MSS	C228T	21.1	95	70	C228T
79	Urinary	51	TMB-high	MSS	C228T	37.1	329	40	C228T
80	Urinary	169	TMB-low	MSS	C228T	30.1	332	80	C228T
81	Urinary	30	TMB-low	MSS	C228T	27.9	219	70	C228T
82	Urinary	15	TMB-low	MSS	C228T	18.1	205	40	C228T
83	Urinary	27	TMB-low	MSS	C228T	32.1	545	40	C228T
84	Urinary	39	TMB-low	MSS	C228T	18.1	276	70	C228T
85	Brain	51	TMB-low	MSS	C228T	51.6	31	70	C228T
86	Colon	64	TMB-high	MSS	C228T	28.3	152	40	C228T
87	Colon	76	TMB-low	MSS	C228T	29.4	286	50	C228T
88	Colon	87	TMB-low	MSS	C228T	31.8	63	90	C228T
89	Colon	61	TMB-high	MSS	C228T	34.3	429	60	C228T
90	Colon	18	TMB-low	MSS	C228A	38.8	268	40	C228A
91	Colon	30	TMB-low	MSS	C228T	38.9	779	30	C228T
92	Colon	42	TMB-low	MSS	C228A	53.2	139	60	C228A
93	Head and neck	54	TMB-low	MSS	C228T	25.3	95	20	C228T
94	Head and neck	66	TMB-low	MSS	C228T	31.6	493	70	C228T
95	Head and neck	77	TMB-low	MSS	C250T	85.1	215	50	C250T
96	Lung	90	TMB-low	MSS	C228T	20.2	134	60	C228T
97	Lung	37	TMB-high	MSS	C228T	26.2	84	40	C228T
98	Sarcoma	26	TMB-low	MSS	C228T	50.0	118	90	C228T
99	Sarcoma	14	TMB-low	MSS	C228T	56.6	53	80	C228T
100	Sarcoma	37	TMB-low	MSS	C250T	67.5	437	50	C250T
101	Sarcoma	45	TMB-low	MSS	C228T	70.9	117	25	C228T
102	Skin	136	TMB-low	MSS	C228T	37.8	394	20	C228T
103	Thyroid	163	TMB-low	MSS	C228T	45.6	204	80	C228T

*NGS* next-generation sequencing, *TERT* telomerase reverse transcriptase, *VAF* variant allele frequency, *TD* total read depth, *TV* tumor volume

**Fig. 1:**
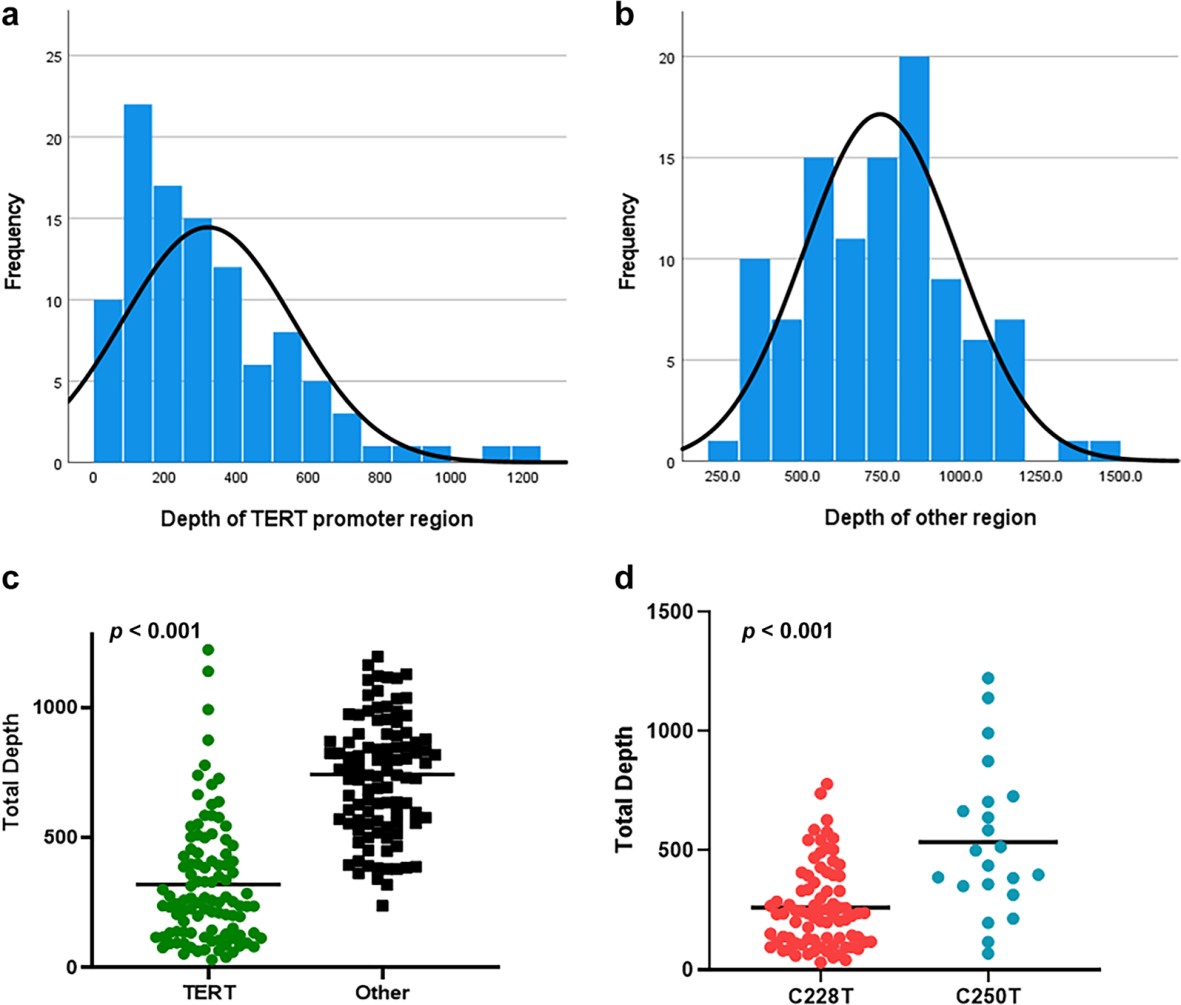
The average depth of sequencing coverage in the telomerase reverse transcriptase (*TERT*) promoter region **a** and other genes **b**. There was a statistically significant decrease in the sequencing read depth in the *TERT* promoter region than the other genes **c**. Sequencing read depth was significantly higher at C250 compared to C228 **d**

With NGS tests, the average sequencing read depth was 300, which was higher than the depth requirements (≥ 150). The average read depth of the *TERT* promoter region was 320.4 (range, 31–1223; median, 254), which was significantly lower than that of the other genes (mean, 743.5; range, 238–1416; median, 757) (*p* < 0.001) (Fig. 1a–c). The average sequencing read depth of the *TERT* promoter regions at C228 and C250 were 261.73 ± 19.13 (range, 31–779; median, 236.0) and 536.41 ± 66.73 (range, 69–1223; median, 468.50), respectively. Interestingly, NGS read depth was higher at C250 compared to C228 with statistical significance (*p* < 0.001) (Fig. 1d). The average VAFs in the C228T and C250T mutations of the *TERT* promoter region were 31.8% (range, 7.7–70.9%) and 32.0% (range, 8.7–85.1%), respectively.

### iTERT PCR and Sanger sequencing

**Fig. 2:**
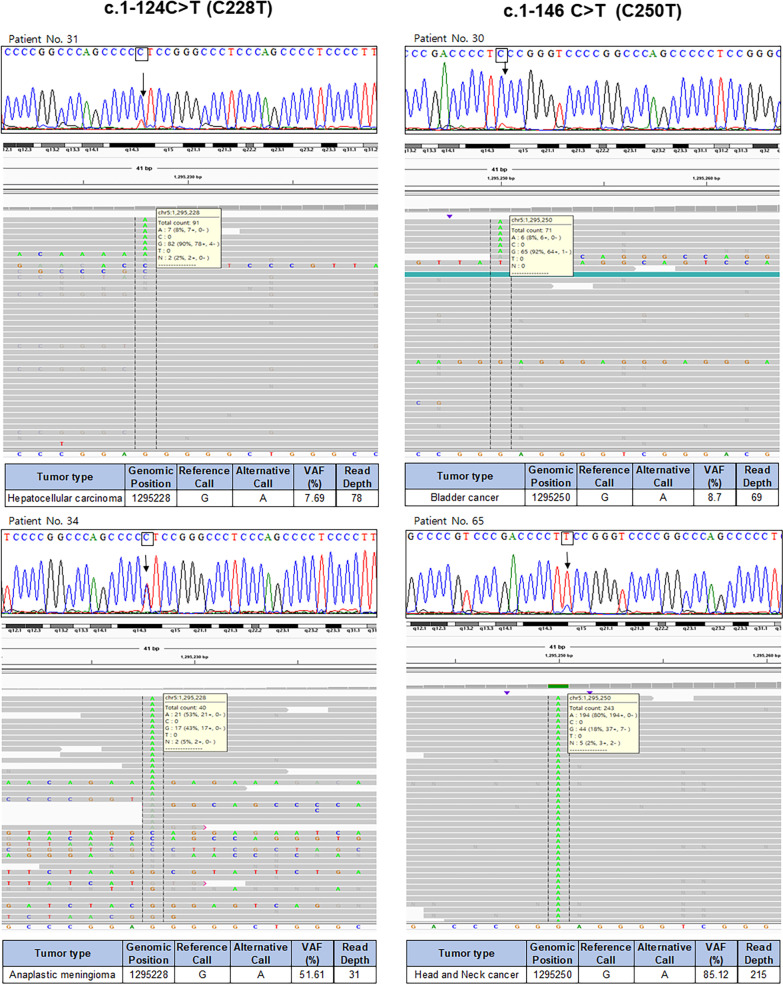
Results of iTERT polymerase chain reaction and Sanger sequencing in the representative cases. According to the variant allele frequencies of the *TERT* promoter mutation, there was good correlation among the heights of the mutant peaks

In 103 cases harboring the *TERT* promoter mutations, iTERT PCR and Sanger sequencing were performed using the same DNA left over after the NGS test. The iTERT PCR test showed 100% sensitivity and specificity for the detection of *TERT* promoter mutations and achieved 100% positive predictive value (PPV) and NPV. The peak heights of the wild-type and mutant alleles detected by Sanger sequencing varied and correlated very well with the VAFs detected using NGS (Fig. 2). Although the mean read depths were relatively smaller in the *TERT* promoter region than in the other regions, we found that the peak heights of mutant alleles in Sanger sequencing correlated well with the VAFs, suggesting that read depths have very little effects on the detection of *TERT* promoter mutations. In addition to the validation of NGS results with Sanger sequencing in the *TERT* promoter region, we also established the efficacy of the iTERT PCR kit.

## Discussion

Two hotspot mutations, C228T and C250T, in the *TERT* promoter region have been proposed as novel mechanisms for the activation of telomerase in malignant cells, and act as important biomarkers for predicting aggressive clinical behavior in various types of cancer [9]. However, the GC-rich sequences within the *TERT* promoter region make their DNA less amenable to PCR amplification. In the present study, we used the commercially available iTERT PCR kit to simultaneously validate the NGS results and explore the analytical sensitivity of the PCR kit. In 103 samples diagnosed with hotspot mutations in the *TERT* promoter region using NGS tests, the same DNA was also tested with the iTERT PCR kit, which verified the presence of the same mutations with 100% agreement. Although the read depth of the *TERT* promoter region was smaller than that of other genes, the peak heights of mutant alleles in Sanger sequencing correlated with the VAFs of the NGS test, suggesting that the read depth has little impact on the detection of *TERT* promoter mutations.

Telomeres are composed of "TTAGGG" repeats at the end of chromosomes and the telomere length plays a critical role in multiple human diseases, including cancer [9]. The *TERT* promoter mutations were found to be the most common point mutations in several types of cancer, including 60–100% of glioblastoma [5, 10, 32, 33], 22–71% of melanoma [4, 15, 34], 29–100% of bladder cancer [3, 35–38], and 29–65% of hepatocellular carcinoma [39–41] cases (Table 2). To date, the C228T and C250T hotspot mutations have been identified in over 50 distinct types of cancer, and they are responsible for the activation of the *TERT* promoter region and *TERT* gene transcription [3, 4].

**Table 2:** Prevalence and distribution of TERT mutations in cancer genomes. The prevalence of TERT mutations in given as percentage and as total number of cases

Cancer type	Our study			Prevalence of mutations in published literatures
	Prevalence of mutations	c.1-124C>T (C228T)	c.1-146 C>T (C250T)	
Urinary bladder	31/47 (66.0%)	24/31 (77.4%)	7/31 (22.6%)	29–100% [3, 6, 7, 35–38, 51]
Pancreatobiliary	19/127 (15.0%)	16/19 (84.2%)	3/19 (15.8%)	0–7% [5, 9, 42, 52]
Liver	22/41 (53.7%)	19/22 (86.4%)	3/22 (13.6%)	29–65% [35, 39, 40]
Melanoma	12/90 (13.2%)	5/12 (41.7%)	7/12 (58.3%)	60–100% [4, 15, 34]

Interestingly, we found that NGS read depth was higher at C250 compared to C228 with statistical significance although GC contents around C228 and C250 were similar (76.9% and 78.3%) and the exact molecular mechanism underlying our results are unknown. TERT promoter mutations, C228T and C250T, were heterozygous and mutually exclusive, but both mutations result in the generation of an 11-bp identical sequence, 5′-CCCCTTCCGGG-3′. Although low read depth of C228T TERT promoter mutation, we confirmed same Sanger sequencing results.

In the present study, we detected the *TERT* promoter mutations in 5.1% of all tested cases by NGS and the majority of these mutations were C228T and C250T. We also identified two C228A mutations from colon cancer samples. The *TERT* promoter mutations were mainly detected in urinary bladder cancer (66%), hepatocellular carcinoma (54%), pancreato-biliary cancer (15%), and malignant melanoma (13%), and the overall incidence was similar to that reported previously [3, 6–8, 35, 42]. As most of the patients whose samples were used for NGS exhibited advanced stages of the disease with aggressive tumor behavior [7], we did not compare the prognostic differences between the patients with and without *TERT* promoter mutations in the present study. The clinicopathological characteristics of the *TERT* promoter mutations in brain [27] and thyroid tumors [43] have been previously reported by researchers at our institute.

To identify any problems associated with the amplification of GC-rich genes (and/or using GC-rich primers) [26, 44, 45], we focused on the read depth of the NGS test as well as the performance of the commercially available PCR kit in the present study. We found that although the read depth was small in the GC-rich *TERT* promoter region, mutations were detected in the samples by NGS and these results were further validated by Sanger sequencing. It is well known that the sensitivity of different NGS workflows can vary between clinical laboratories, particularly based on the bioinformatic pipeline used and the types of variants that the pipeline is designed and validated to detect. Therefore, carefully evaluating the coverage of NGS remains vital [46]. For many clinical laboratories adopting NGS as a diagnostic platform, detection of low-VAF somatic mutations is a challenge [47]. Even at a high read depth, NGS shows a rapid drop in detection accuracy of low-VAF somatic mutations [48–50].

In the present study, although the average read depth of the *TERT* promoter region was significantly lower than that of the other genes, we observed that the average VAFs in the C228T and C250T mutations of the *TERT* promoter region were more than 30% and the lowest VAF was 7.7%. These results suggest that mutations in the *TERT* promoter region are shared by many tumor cells and make the *TERT* promoter mutation accurate with relatively low read depth in the GC-rich *TERT* promoter region in NGS. Moreover, high VAFs in the *TERT* promoter mutation enabled high PPV and NPV using the iTERT PCR kit.

Several cancers are reported to harbor frequent mutations in the *TERT* promoter region [7]. Moreover, the simple and inexpensive iTERT PCR kit successfully demonstrated the *TERT* promoter mutations detected by NGS in all tested cases, even with miniscule amounts (~ 10 ng/μl) of DNA (Table 3). Therefore, we validated the NGS results with the gold standard PCR test and found that the iTERT PCR test is sensitive for the identification of the *TERT* promoter mutations in solid cancers. Based on these observations, we can suggest the iTERT PCR test as a simple, cheap, easily accessible, and effective alternative to NGS that can be widely used for the detection of *TERT* promoter mutations in diagnostic laboratories.

**Table 3:** Comparison of iTERT PCR with NGS

	NGS	iTERT PCR
Quality of DNA	Limited by damaged DNA in certain cases Needed high-quality DNA	Rarely limited by damaged DNA
Quantity of DNA	Needed the amount of DNA required for downstream NGS preparation steps (50 ~ 120 ng)	Relatively 'small' amount of DNA is required (< 50 ng)
Test time	Requires more time for the preparation of library preparation (2 days)	Time-saving and easy PCR preparation (< 3 h)
Costs (per case)	£570	£30
Interpretation	Very complex, and its interpretation requires expert bioinformatics assistance	Easy to analyze PCR-Sanger sequencing results

## Data Availability

Reference genome (hg19) used in this study can be obtained from the UCSC databases (https://hgdownload.soe.ucsc.edu/). The datasets generated during and/or analyzed during the current study are available from the corresponding author upon reasonable request.

## Abbreviations

NGS: Next-generation sequencing
TERT: Telomerase reverse transcriptase
PCR: Polymerase chain reaction
TSO: TruSight Oncology
VAF: Variant allele frequency
TD: Total read depth
TV: Tumor volume
